# Optimizing Pain Control and Function in Patients With Adhesive Capsulitis by Choosing the Best Injection Site

**DOI:** 10.31486/toj.22.0014

**Published:** 2022

**Authors:** Sherilyn DeStefano, Lauren Oberle, Brian Donohoe, Yuka Kobayashi, Andrew W. Gottschalk

**Affiliations:** ^1^ Department of Family Medicine, Division of Sports Medicine, University of California Los Angeles, Los Angeles, CA; ^2^ Department of Orthopedic Surgery, Department of Family Medicine, Medical College of Wisconsin, Milwaukee, WI; ^3^ Department of Orthopedics, Sports Medicine Institute, Ochsner Clinic Foundation, New Orleans, LA

## CASE PRESENTATION

Two patients present to the sports medicine clinic with shoulder pain and limited range of motion. A 51-year-old healthy female rower presents 5 months after developing left shoulder pain. She had no specific inciting injury, although she increased overhead workouts with dumbbells prior to the onset of pain. She describes soreness that started in the left biceps and then shifted to the lateral shoulder. The other patient, a 61-year-old male golfer with a history of hyperlipidemia and hypertension, developed right shoulder pain also after increasing overhead exercises with weights 3 months prior to presentation. His pain is most significant with sleeping on the right side, abducting the right arm, and lifting heavy objects. Both patients have limited shoulder flexion, abduction, and internal rotation despite 8 weeks of physical therapy.

## BACKGROUND

Adhesive capsulitis, commonly referred to as frozen shoulder, is a relatively common condition that results in significant pain and limitation of active and passive range of motion of the glenohumeral joint.^1-3^ It has been estimated to affect less than 1% to 5% of the general population but up to 20% of patients with diabetes.^1-3^ The condition is thought to be caused by an initial musculotendinous or tenosynovitis inflammatory process that then leads to capsular thickening and adhesion formation, although this historically accepted pathophysiology has started to be questioned.^2,4^ However, both patients in our case presented with a history of increased activities that led to an initial inflammatory process, followed by limited range of motion.

Adhesive capsulitis is characterized by three stages: pain with progressively decreasing range of motion of the glenohumeral joint (freezing stage, lasting 2 to 9 months), stiffness (frozen stage, lasting 4 to 12 months), and, finally, improvement in range of motion with resolution of symptoms (thawing stage, lasting 5 to 42 months).^1-3^ Although adhesive capsulitis is often described as a self-limiting condition that spontaneously resolves after 2 to 3 years, up to 40% of patients experience persistent symptoms, ranging from mild discomfort to permanent disability.^1-3^ Treatment options include anti-inflammatory oral medications, physical therapy, steroid injections, capsular or arthrographic distension, and operative management.^3^ Corticosteroid injections are a common treatment option to treat pain and dysfunction from adhesive capsulitis, and a 2020 meta-analysis showed superior outcomes in short-term pain control, range of motion, and function, as well as longer term functional outcomes with corticosteroid injections compared to no treatment or placebo.^1^ However, studies and clinical care remain heterogeneous regarding the optimal injection location. This review evaluates whether the site of the corticosteroid injection affects pain control and functional outcomes in patients with adhesive capsulitis.

## REVIEW OF EVIDENCE

Multiple studies, including a number of randomized controlled trials, have evaluated the different sites of corticosteroid injections in the treatment of adhesive capsulitis. A systematic review and meta-analysis by Challoumas et al^1^ in 2020 compared pain, using visual analog scale (VAS) pain scores, and function, using validated scores (Shoulder Pain and Disability Index,^5,6^ the American Shoulder and Elbow Surgeons Standardized Shoulder Assessment Form score,^7^ the Constant-Murley Score,^8,9^ and the Shoulder Disability Questionnaire^10^), in patients receiving intra-articular vs subacromial injections for adhesive capsulitis. The outcomes showed that intra-articular steroid injections were superior to subacromial injections for early short-term (2 to 6 weeks) pain control (*P*=0.02) but had similar functional outcomes.^1^ Intra-articular injections had similar late short-term (8 to 12 weeks) pain control and superior late short-term functional outcomes compared to subacromial injections (*P*=0.03).^1^ Midterm (<12 months) results were similar in pain and function between the 2 injection sites. When compared to physical therapy alone, intra-articular steroid injections were superior for early short-term function and late short-term pain; however, no differences were seen in midterm results.^1^ Range of motion was not significantly affected by physical therapy, intra-articular corticosteroid injections, arthrographic distension, or acupuncture, except when intra-articular corticosteroid injection alone was compared to intra-articular injection plus physical therapy which showed that the latter had superior outcomes in the early short-term period only.^1^

Another meta-analysis and systematic review by Shang et al in 2019 also indicated better pain relief measured by VAS scores at 2 to 3 weeks following intra-articular injection compared to subacromial injection (*P*=0.02).^11^ In this review, the corticosteroid selected in 5 studies was 1 mL 40 mg/mL triamcinolone, while 4 studies used 1 mL 40 mg/mL methylprednisolone acetate. In all but 1 study, lidocaine 1% to 2% was mixed with the corticosteroid, with a total volume of 1 to 10 mL injected. Subacromial injections showed greater improvement in internal rotation at 8 to 12 weeks (*P*=0.02), although this difference in improvement did not translate to a difference in function (the American Shoulder and Elbow Surgeons Standardized Shoulder Assessment Form score^7^ and the Constant-Murley Score^8,9^).^11^ On subgroup analysis, no further differences in pain or range of motion were seen at time points up to 24 weeks, and the pooled effect showed no overall differences between the 2 injection sites.^11^ These findings imply that while the 2 injection sites may differ in outcomes at specific time points, improvements do not last longer than the specified time frame.^11^

A 2019 meta-analysis and systematic review by Chen et al included 7 randomized controlled trials that also compared intra-articular to subacromial corticosteroid injections.^2^ The majority of the injections consisted of a combination of 40 mg of triamcinolone and 2% lidocaine, while 1 study used 40 mg of methylprednisolone. The authors found that the intra-articular injections led to improved VAS scores at 1, 2, and 3 months, but no significant differences in range of motion outcomes were seen between the groups.^2^ The authors noted possible bias and reported large heterogeneity between the studies but concluded that the level of evidence was moderate.^2^

The 3 meta-analyses described above reviewed many of the same randomized controlled trials. These studies were heterogeneous regarding image-guided vs landmark-guided injections, stage at which the injection was done (ie, freezing vs frozen vs thawing), and type and amount of steroid and anesthetic injected.

Some data suggest that multisite corticosteroid injections may be more effective than an injection at a single site. Pushpasekaran et al conducted a prospective randomized study comparing a group that received a landmark-guided single posterior injection of 40 mg of methylprednisolone and 2 mL of 2% lidocaine (n=43) to another group that received a landmark-guided injection into 3 sites (posterior, subacromial, and subcoracoid, n=42) with the same mixture diluted with 8 mL normal saline.^12^ The posterior approach was placed “2 cm below and lateral to angle of acromion directed towards coracoid,” subacromial was placed “directed towards acromioclavicular joint through supraspinatus muscle,” and subcoracoid was “obtained by running the needle on subscapularis surface 0.5-1 cm inferior and lateral to coracoid passing towards the lateral third of coracoid,” all of which were completed while the arm was externally rotated during the injection of the mixture.^12^ The Constant-Murley Score^8,9^ was used at baseline and follow-up at 3 weeks, 6 weeks, and 6 months postinjection. Pain scores significantly improved at each follow-up point in both groups, but pain improvement in the 3-site injection group was higher and occurred earlier (*P*<0.0005).^12^ The 3-site injection group also had more improvement in abduction and external rotation than the single-site injection group and reached near-normal scores in all 3 planes at 6-week follow-up.^12^

In the Koraman et al prospective randomized comparative study, the multisite injection group received 2 mL of triamcinolone acetonide (40 mg/mL), 4 mL of bupivacaine (0.5%), and 34 mL of saline solution divided between the glenohumeral joint, posteroinferior capsule, subacromial space, posterosuperior capsule, biceps long head, and coracohumeral ligament.^13^ The single injection group received 1 mL of triamcinolone acetonide (40 mg/mL) and 2 mL of bupivacaine (0.5%) in the glenohumeral joint. Both groups underwent ultrasound-guided injections. The multisite corticosteroid injections resulted in significantly lower VAS scores at 1 month (*P*<0.001), 3 months (*P*<0.001), 6 months (*P*<0.01), and 1 year (*P*=0.012) compared with the single-site injection group, as well as significantly better active and passive range of motion at all time points (*P*<0.05).^13^

Koh published a systematic review in 2016 examining the safety of corticosteroid injections.^3^ The main side effects reported were pain after injection (29.8%), facial flushing (12.3%), and menstrual irregularities (10.1%), with a number needed to harm for steroid injection vs physical therapy of 11.4 for pain, 9.5 for menstrual irregularities, and 7.1 for facial flushing.^3^ No studies reported tendon rupture, a theoretical risk of corticosteroid injections.^3^

Corticosteroids increase blood glucose levels through increased insulin resistance, especially in patients with underlying diabetes or impaired glucose tolerance.^14^ Habib and Miari investigated the effects of corticosteroid injections in patients with well-controlled diabetes and knee osteoarthritis.^15^ The small (n=30) prospective randomized short-term study found that intra-articular knee injections with 20 mg of triamcinolone hexacetonide or 40 mg of triamcinolone acetonide led to significant increases in blood glucose levels, with peak levels at 32.5 hours postinjection when using triamcinolone hexacetonide and 24.5 hours postinjection when using triamcinolone acetonide.^15^ Given this information, all patients should be counseled on the risk of post corticosteroid injection hyperglycemia.

## DISCUSSION

In the treatment of adhesive capsulitis, intra-articular injections appear superior to subacromial injections for short-term pain control, and multisite injections may be superior to single-site injections for pain control and range of motion.^1,2,11-13^ Any injection is likely to be superior to physical therapy alone for short-term pain control.^1^ However, the heterogeneity among the studies should be considered when individualizing a patient's treatment plan.

Overall, corticosteroid injections are well tolerated with infrequent significant side effects. The multisite injection studies show promise but used different injection sites and injectate volumes than the studies in the meta-analyses. Also, a limitation of both multisite injection studies is the absence of a control group.^12,13^

Some primary care physicians may not feel comfortable giving intra-articular injections, and data suggest that landmark-guided intra-articular injections are less accurate than image-guided injections (72.5% vs 92.5%, *P*=0.025).^16^ In contrast, landmark-guided subacromial injections appear to have the same accuracy as image-guided injections (65.0% vs 70.0%, *P*>0.05).^16^ In situations when timely referral to a sports medicine or orthopedic specialist is not available for intra-articular or multisite injection, a landmark-guided subacromial injection may be a reasonable first step for pain control in patients with adhesive capsulitis.

## CASE RESOLUTION

Both patients were found to be in the freezing stage of adhesive capsulitis and received a corticosteroid injection in conjunction with physical therapy. The 51-year-old female received a single-site landmark-guided subacromial injection of 1 mL of 40 mg/mL triamcinolone acetonide and 4 mL of 1% lidocaine without epinephrine, while the 61-year-old male received a landmark-guided glenohumeral intra-articular injection with 1 mL of betamethasone 6 mg/1 mL and 4 mL 1% lidocaine without epinephrine. Post intra-articular corticosteroid injection, the male patient had improved pain and function, measured using the Shoulder Pain and Disability Index, and restoration of uninterrupted sleep at 2 weeks and 10 weeks. At 11 weeks after the injection, his range of motion had improved by approximately 15° of forward flexion and 5° of both internal and external rotation. Post subacromial injection, the female patient had improvement in pain and function, also measured using the Shoulder Pain and Disability Index, at 4 weeks, although she reported that the relief decreased at 8 weeks postinjection. She did not have improvement in range of motion.

## Notes

*This article meets the Accreditation Council for Graduate Medical Education and the American Board of Medical Specialties Maintenance of Certification competencies for Patient Care and Medical Knowledge.*

## References

[1] Challoumas D, Biddle M, McLean M, Millar NL. Comparison of treatments for frozen shoulder: a systematic review and meta-analysis. JAMA Netw Open. 2020;3(12):e2029581. doi: 10.1001/jamanetworkopen.2020.29581 33326025 PMC7745103

[2] Chen R, Jiang C, Huang G. Comparison of intra-articular and subacromial corticosteroid injection in frozen shoulder: A meta-analysis of randomized controlled trials. Int J Surg. 2019;68:92-103. doi: 10.1016/j.ijsu.2019.06.008 31255719

[3] Koh KH. Corticosteroid injection for adhesive capsulitis in primary care: a systematic review of randomised clinical trials. Singapore Med J. 2016;57(12):646-657. doi: 10.11622/smedj.2016146 27570870 PMC5165171

[4] Wong CK, Levine WN, Deo K, et al. Natural history of frozen shoulder: fact or fiction? A systematic review. Physiotherapy. 2017;103(1):40-47. doi: 10.1016/j.physio.2016.05.009 27641499

[5] Staples MP, Forbes A, Green S, Buchbinder R. Shoulder-specific disability measures showed acceptable construct validity and responsiveness. J Clin Epidemiol. 2010;63(2):163-170. doi: 10.1016/j.jclinepi.2009.03.023 19683414

[6] Tveitå EK, Ekeberg OM, Juel NG, Bautz-Holter E. Responsiveness of the shoulder pain and disability index in patients with adhesive capsulitis. BMC Musculoskelet Disord. 2008;9:161. doi: 10.1186/1471-2474-9-161 19055757 PMC2633286

[7] Michener LA, McClure PW, Sennett BJ. American Shoulder and Elbow Surgeons Standardized Shoulder Assessment Form, patient self-report section: reliability, validity, and responsiveness. J Shoulder Elbow Surg. 2002;11(6):587-594. doi: 10.1067/mse.2002.127096 12469084

[8] Cook KF, Roddey TS, Olson SL, Gartsman GM, Valenzuela FF, Hanten WP. Reliability by surgical status of self-reported outcomes in patients who have shoulder pathologies. J Orthop Sports Phys Ther. 2002;32(7):336-346. doi: 10.2519/jospt.2002.32.7.336 12113468

[9] Rocourt MH, Radlinger L, Kalberer F, et al. Evaluation of intratester and intertester reliability of the Constant-Murley shoulder assessment. J Shoulder Elbow Surg. 2008;17(2):364-369. doi: 10.1016/j.jse.2007.06.024 18329560

[10] van der Windt DA, van der Heijden GJ, de Winter AF, Koes BW, Devillé W, Bouter LM. The responsiveness of the shoulder disability questionnaire. Ann Rheum Dis. 1998;57(2):82-87. doi: 10.1136/ard.57.2.82 9613336 PMC1752535

[11] Shang X, Zhang Z, Pan X, Li J, Li Q. Intra-articular versus subacromial corticosteroid injection for the treatment of adhesive capsulitis: a meta-analysis and systematic review. Biomed Res Int. 2019;2019:1274790. doi: 10.1155/2019/1274790 31737653 PMC6815644

[12] Pushpasekaran N, Kumar N, Chopra RK, Borah D, Arora S. Thawing frozen shoulder by steroid injection. J Orthop Surg (Hong Kong). 2017;25(1):2309499016684470. doi: 10.1177/2309499016684470 28142344

[13] Koraman E, Turkmen I, Uygur E, Poyanlı O. A multisite injection is more effective than a single glenohumeral injection of corticosteroid in the treatment of primary frozen shoulder: a randomized controlled trial. Arthroscopy. 2021;37(7):2031-2040. doi: 10.1016/j.arthro.2021.01.069 33581295

[14] Tamez-Pérez HE, Quintanilla-Flores DL, Rodríguez-Gutiérrez R, González-González JG, Tamez-Peña AL. Steroid hyperglycemia: Prevalence, early detection and therapeutic recommendations: a narrative review. World J Diabetes. 2015;6(8):1073-1081. doi: 10.4239/wjd.v6.i8.1073 26240704 PMC4515447

[15] Habib GS, Miari W. The effect of intra-articular triamcinolone preparations on blood glucose levels in diabetic patients: a controlled study. J Clin Rheumatol. 2011;17(6):302-305. doi: 10.1097/RHU.0b013e31822acd7c 21869712

[16] Aly AR, Rajasekaran S, Ashworth N. Ultrasound-guided shoulder girdle injections are more accurate and more effective than landmark-guided injections: a systematic review and meta-analysis. Br J Sports Med. 2015;49(16):1042-1049. doi: 10.1136/bjsports-2014-093573 25403682

